# Environmental enrichment with nylon gnaw sticks introduces variation in Sprague Dawley rat immune and lower gastrointestinal parameters with differences between sexes

**DOI:** 10.1186/s42523-024-00369-6

**Published:** 2025-01-31

**Authors:** Mark Wulczynski, Stephen P. J. Brooks, Judy Green, Fernando Matias, Martin Kalmokoff, Julia M. Green-Johnson, Sandra T. Clarke

**Affiliations:** 1 Applied Bioscience Graduate Program, Faculty of Science, Ontario Tech University, Oshawa, ON Canada; 2 Bureau of Nutritional Sciences, Health Canada, Ottawa, ON Canada; 3 Kentville Research and Development Centre, Agriculture and Agri-Food Canada, Kentville, NS Canada; 4 Guelph Research and Development Centre, Agriculture and Agri-Food Canada, Guelph, ON Canada

**Keywords:** Gnaw sticks, Environmental enrichment, Rat, Gut microbiome, Cytokines, Nutrition, Immune system, Short chain fatty acids, Sex-based differences

## Abstract

**Background:**

Environmental enrichment (EE) is commonly included as an important component of animal housing to promote well being of laboratory animals; however, much remains to be learned about the impact of chewable forms of EE on experimental outcomes in the context of nutritional and microbiome-related studies, and whether outcomes differ between sexes. In the present study, nylon chew bones (gnaw sticks, GS) were evaluated for their effects on fermentation profiles, microbial community structure, and cytokine profiles of gastrointestinal and systemic tissues in pair-housed female and male Sprague Dawley (SD) rats.

**Results:**

Food consumption and weight gain were not significantly altered by access to GS. Cecal short-chain fatty acid and branched-chain fatty acid profiles significantly differed between sexes in rats with access to GS, and alpha diversity of the microbiome decreased in females provided GS. Sex-related tissue cytokine profiles also significantly differed between rats with and without access to GS.

**Conclusions:**

These findings indicate that including GS can influence microbiota and immune-related parameters, in a sex dependent manner. This shows that environmental enrichment strategies need to be clearly reported in publications to properly evaluate and compare experimental results, especially with respect to the use of chewable EE in the context of studies examining diet, microbiome and immune parameters.

**Supplementary Information:**

The online version contains supplementary material available at 10.1186/s42523-024-00369-6.

## Background

Environmental enrichment (EE) is recognized as essential for optimal animal welfare, but little is known about its potential impact on nutrition-related outcomes. Comparisons against control groups indicate EE provides rodents with both sensory and motor stimulation that promote species-typical behaviors and physiological well-being [1]. The inclusion of EE also improves the reproducibility of experimental results, as it has long been established that relaxed, alert and busy animals have more consistent behaviours and sex hormone concentrations [2].The term EE encompasses a range of strategies and materials rather than a standardized approach, posing challenges in comparing the impact of EE between studies. Typical examples of EE are nesting materials and structures but can also include toys to stimulate activity and chewable items such as gnaw sticks (GS). Recent reviews of EE have highlighted current issues such as the need for consistent terminology in describing EE, interpreting the impact of EE in complex environments, and the importance of considering sex-based biological differences in choosing EE [3–6]. Many studies of EE outcomes focus on cage modifications, husbandry strategies, social housing, bedding and nesting materials [3, 6].

In the context of nutrition research, immunological and endocrinological outcomes are of particular interest. Recent findings in rats indicate that loss of EE led to increased food consumption and to decreased active behaviour in response to stress in a forced swim test [7, 8]. Removal of EE also affected Hypothalamic Pituitary Adrenal (HPA) axis responses to acute restraint stress as determined by plasma corticosterone (CORT) levels, with sex-related differences in CORT response kinetics [7]. Several studies indicate differences between sexes in the outcomes of EE at the behavioural, neurological and endocrine level. For example, sex-specific differences in the effects of closed nestbox rearing are seen in the levels of neurotrophic factors brain-derived neurotrophic factor (BDNF) and glial-derived neurotrophic factor (GDNF) in Long-Evans rat pups in a model of hypoxic-ischemic (HI) encephalopathy [9], with HI-injured males showing higher BDNF levels in the striatum, and HI-injured females showing higher GDNF levels in the hippocampus. Differences in EE impact between sexes have been observed for varied behavioural measures including navigation-based tasks [10] and stress-associated behavioural changes associated with maternal deprivation [11]. Mechanisms underlying these differences are beginning to be elucidated. Several studies focusing on EE in the context of early life stress (ELS) induced by maternal deprivation report differences between sexes in the ability of EE to reverse stress-associated changes at the biochemical, molecular and epigenetic level, including oxidative stress parameters [11], histone deacetylase and DNA methyltransferase activity in the hippocampus and prefrontal cortex [12], and hypothalamic miRNA expression [13].

While numerous studies have examined behavioural effects of EE, less is known about the impact on the immune system or on the gut microbiota, especially in relation to diet or to sex-based differences. Effects of EE on immune measures in laboratory rodents include increases in Natural Killer (NK) cell activity, macrophage and lymphocyte chemotaxis, macrophage phagocytic activity, and production of interleukin-2 (IL-2) and tumour necrosis factor-α (TNF-α) in aged mice provided with novel objects [6, 14]. Sprague Dawley (SD) rats housed in multilevel caging systems show lower neutrophil: lymphocyte ratios than rats with access restricted to only one cage level, suggesting rats with restricted access were distressed [15]. Inclusion of novel objects as EE has also been reported to decrease expression of the pro-inflammatory cytokines TNF-α and IL-1β, and to attenuate stress-induced pro-inflammatory cytokine production in mice and rats (reviewed in [6, 16]). The presence of EE in the form of ladders, jars and nesting material has been reported to decrease numbers of immature thymocytes (double-positive CD4^+^CD8^+^ thymocytes) in female Swiss Webster and BALB/c mice compared to mice with only standard cages and bedding. In addition, female Swiss Webster mice with this type of EE have higher levels of the regulatory cytokines IL-10 and IL-4 than those without EE [17], suggesting potential differences in the impact of EE between sexes. Cage enrichment with materials enabling mice to create three-dimensional nests has been reported to be associated with increased spleen mass, splenic B and T cell numbers and secondary responses to influenza vaccination in male BALB/c mice, findings negatively associated with effects of EE on corticosteroid production [18]. Additionally, EE impacts exploratory behaviour (with access to visual stimuli, running wheels, plastic tubing, rubber balls) [19], aggression between animals (with access to nesting material and shelters) [20, 21] and time spent sleeping (with access to nesting material and plastic tubes) [22] amongst several strains of mice. The effect of EE on neurological development, which has been most frequently studied, influences stress and cognitive function through effects on development of the hippocampus, prefrontal cortex, and amygdala [23]. In contrast, relatively few studies have compared or evaluated the impact of EE on immunological, nutritional, microbiological or toxicological measures, or compared differences in impact on these measures between sexes.

Recent studies point to the importance of examining differences in the impact of EE between sexes with respect to neuroimmune interactions (reviewed in [6]). Pavlova et al. compared effects of long-term EE on behaviour of female and male rats exposed to neonatal pro-inflammatory challenge induced by LPS, using several tests to evaluate effects on anxiety, depressive and fear-associated behaviours [24]. While rats housed under EE conditions (three-tier cages with ladders, wheel, toys, hammocks and burrowing material) showed heightened search and motor activity compared to rats housed in standard conditions, differences between males and females were seen in the extent to which EE reduced anxiety, depressive-like behaviour, corticosterone responses to forced swim stress and basal serum IL-1β levels.

Several studies have examined effects of EE on varied aspects of maternal-related behavioural and immune measures in rats, including EE in the context of maternal separation to induce ELS [13, 25] as well as EE impact on mothers and offspring in maternal immune activation (MIA) [26–28]. EE provided as toys, tubes, chew bones, and Nestlets has been reported to influence SD rat litter size, nursing behaviour, milk triglyceride levels and maternal milk microbiome diversity as well as social behaviour of offspring, supporting the importance of EE in the maternal environment [29]. In models using maternal separation to induce ELS in rats, EE provided as bedding, toys, tunnels and platforms prevented ELS-induced elevation of the pro-inflammatory cytokine TNF-α in males, but not in females, while preventing cognitive dysfunction in both sexes [25]. Protective effects of EE in LPS-induced MIA in rats with respect to neurological, endocrine and developmental effects associated with altered cognitive and behavioural activity of offspring have also been shown [26–28]. EE attenuated several outcomes of LPS-induced MIA at the placental level and in offspring, reflecting changes in expression of genes associated with synaptic plasticity, as well as epigenetic changes [26, 27]. EE diminished MIA-induced elevation of circulating corticosterone in female offspring, and ameliorated MIA-induced downregulation of *Eaat2*, (a marker of synaptic plasticity) in female offspring only, indicating sexual dimorphism in the responses to MIA and EE [26].

Overall, these findings suggest that a lack of EE standardization among experiments could contribute to differences in outcomes among studies, reflecting differences in EE forms, timing of EE exposure, and duration of access [30]. As part of our investigation into the impact of EE on gastro-intestinal related outcomes, we previously showed that housing conditions influence fecal bacterial community structure and tissue cytokine profiles in rats, and demonstrated that bedding material can significantly impact microbial activity and immune measures [31]. In the present study, our focus was on nylon chew bones (GS), which are often provided to rodents as an additional EE measure, and may act as a source of indigestible particulates. To our knowledge, no studies have evaluated the effect of GS on immunological parameters related to fermentation events in the lower bowel or analyzed sex-based differences in this context. This is important since particulate, indigestible matter has been shown to alter transit time and may affect the amount of material entering the lower bowel [32]. While numerous studies have examined the impact of factors including housing conditions, transport, bedding and diet on the gut microbiota of laboratory rodents, much remains to be explored regarding EE and the role of sex-based differences in the context of nutrition research (reviewed in [33]).

Currently little is known about the impact of GS, a type of EE that is especially relevant in studies focused on nutrition, diet and the gut microbiota. In this study, we evaluated the impact of GS on gut microbial community parameters in female and male SD rats, including community diversity, short chain fatty acid (SCFA) and branched chain fatty acid (BCFA) production, standard liver and kidney biochemistry measures, and on mucosal and systemic tissue cytokine profiles. Furthermore, we examined how inclusion of this type of EE influenced experimental outcomes relevant for studies of effects of diet on the gut microbiota and the immune system, and explore potential differences in impact between sexes.

## Materials and methods

### Experimental design and animal maintenance

This study was carried out in accordance with the guidelines of the Canadian Council for Laboratory Animals and the experimental protocol was approved by the animal care committees at both Health Canada and Ontario Tech University. This experiment utilized 28-30d SD rats (1 week post weaning, Charles River Laboratories, QC), with *n* = 16 males and *n* = 16 females. Rats were pair-housed by matching sex in SealSafe-Plus plastic cages (Techniplast, Toronto, ON). The bottoms of the cages were lined with an iso-Pad™ (Braintree Scientific Inc., Braintree, MA, USA) along with TechElite paper (Shepherd Specialty Papers, Milford, NJ, USA) and covered by a wire grid to create flooring, along with a solid 4 × 9-inch stainless steel resting plate. Cages were attached to a Smart Flow Air Handling Unit (Techniplast) that provided a constant flow of HEPA-filtered air operating in containment mode. Vivarium conditions were held on a 12 h light/dark cycle at 21 °C and 40% humidity, and rats had free access to AIN-93G diet and reverse osmosis-treated water. EE (glass balls, background radio music and stainless steel shelter) was provided for all rats. Half of the rats (*n* = 8 males and females) were also provided with GS (petite green whole nylon bones, Bio- Serv^®^, Flemington NJ), referred to as “gnaw sticks”. The trial was run over a total of 9 weeks, which included an initial 2 week acclimatization period followed by a 7 week feeding trial, with the final 2 weeks of feeding dedicated as a balance study to measure total input/output. During the second week of acclimation, all rats received tail tattoos with identification numbers.

### Animal health status and tissue collection

Rats were monitored daily for changes in health status, which included behavioural changes (restlessness, altered food consumption, sensitivity to handling, sensitivity to noise and light) and evidence of diarrhea. Following the 9-week trial, rats were anaesthetized with isoflurane (Aerane™; Boxter Healthcare, Deerfield, IL, USA) and exsanguinated *via* cardiac puncture. Blood was collected in BD Vacutainer SST™ tubes (Becton, Dickson and Company, Franklin Lakes, NJ) for serum separation. During necropsy, tissues (spleen, liver, mesenteric lymph nodes (MLN), ileal Peyer’s patches, ileum, cecum and distal colon) as well as cecal and colonic contents were collected, weights recorded, snap frozen and stored at -80 °C until processing.

### Metabolites and automated biochemical analyses

Fermentation end products, including SCFA and BCFA, were measured by gas chromatography [34]. Briefly, approximately 1 g of colonic pellets or cecal content material was homogenized in acid, centrifuged to remove insoluble materials, filtered (0.25 micron syringe), and injected onto a 60 m × 0.25 mm, I.D. 0.25 μm film thickness Nukol column (Supleco-Sigma-Aldrich, Mississauga ON). Analysis was performed on an Agilent 6890 gas chromatograph (Agilent Technologies Canada Inc., Mississauga ON) and analysed using the Agilent associated MSD Chemstation software.

Non-fasting serum samples collected at necropsy were analyzed for total blood urea nitrogen (BUN), and concentrations of liver enzymes aspartate aminotransferase (AST), alanine aminotransferase (ALT), alkaline phosphate (ALP) and lactate dehydrogenase (LDH) using an ABX Pentra 400 automated clinical chemistry analyzer with APX Pentra test kits (Horiba Canada Inc., Burlington, ON). BUN was measured using the urease-glutamate dehydrogenase method [35] with urea CP test kits (Horiba Canada Inc., Burlington, ON). The liver-related enzymes were measured by specific reaction pathways as previously described [31].

### DNA isolation and sequence analysis

Cecal samples were ground in liquid nitrogen [36] and the community DNA was isolated using the QIAgen faecal DNA isolation kit carried out according to the manufacturer’s procedure for difficult to lyse bacteria (QIAgen, Toronto, ON). Isolated DNA was quantified by spectrophotometry and stored frozen at -20 °C. The V4 region of the community bacterial 16 S rRNA genes were sequenced using manufacturer recommended protocols (Illumina, San Diego, CA) with primers and conditions as previously described [37]. Amplicons were bi-directionally sequenced, quality filtered, assembled, and trimmed to give a final sequence of about 240 bp. Sequences were processed using MOTHUR [38]. Quantitative PCR was used to estimate total 16 S rRNA content using the universal primers HDA1 and HDA2 [39].

### Tissue homogenization and cytokine quantification

Frozen tissues were mechanically homogenized using a VWR^®^200 homogenizer (VWR International, Radnor, PA, USA) in ice-cold immunoprecipitation buffer (50 mM NaH2PO4, 100 mM Na2PO4, 0.1% sodium dodecyl sulfate, 0.5% NaCl, 1% Triton X-100, 5 mg/mL sodium deoxycholate) [40], with 1% protease inhibitor cocktail (Sigma Aldrich, St. Louis, MO, USA). Samples were then centrifuged at 16,400 rcf for 30 min at 4 °C, and supernatants were collected and frozen at -80 °C until analysis. Tissue cytokine concentrations (spleen, liver, MLN, ileum, cecum and distal colon) were determined using DuoSet ELISA kits from R&D Systems (Minneapolis, MN, USA) using the manufacturer’s standard protocol and recommended reagent concentrations with 96-well high binding Microlon 600 ELISA plates (Greiner Bio-One, NC, USA). The tissue homogenates were analyzed for pro-inflammatory cytokines TNF-α, interferon-γ (IFN-γ), IL-1β, IL-6, and IL-17 F, chemokines cytokine-induced neutrophil chemoattractant 1 (CINC-1) and soluble intercellular adhesion molecule-1 (sICAM-1), and regulatory cytokines IL-4, IL-10, and transforming growth factor-beta 1 (TGF-β1) in both active and total forms. Cytokine concentrations were analyzed at a wavelength of 450 nm using a Synergy HTTR microplate reader (BioTek Instrumentation, VT, USA). The cytokine concentrations of ileal Peyer’s patches were measured using the ProcartaPlex Rat Th Complete Panel 14 plex assay (Thermo Fisher Scientific, Waltham, MA, USA) according to the manufacturer’s instructions and analyzed using a Luminex MAGPIX^®^ (Luminex, Austin, TX, USA).

### Statistics

Sample size calculations indicated that 8 animals/diet were required to determine a physiologically significant difference of about 1 ng/g tissue of the cytokines of interest (power of 80% with a type 1 error rate of 0.05). This was based on a previously observed 30% coefficient of variation for measurements of immunological parameters in male rats. While pair housed, the measures are from individual samples.

OTUs occurring ≥ 5 times or OTUS occurring between 3 and 5 times with greater than 95% matches to a sequence in the Silva database were included in the analysis. This ensured that sequencing errors did not influence OTU identification. Compositional 16 S rRNA gene abundance data was analysed using PC-ORD v7.07 [41] (Wild Blueberry Media, Corvallis OR) for ecological data [42]. Data was visualized using non-metric multidimensional scaling (NMS) since it is less sensitive to deviations from normality [41]. NMS ordination was performed in auto-pilot mode (maximum 6-axis, 500 iterations) using an Euclidean distance measure (PC-ORD v7.07). Axis ordination was confirmed using Principal Coordinates Analysis to obtain data on variance attributable to axes.

The joint plot function in PC-ORD was used to identify the primary sources of bacterial community variation at the family and phylotype levels, and to assess the impact of various parameters on community change (SCFA, BCFA, protein source, BUN, carbohydrate source) using a cut-off of r^2^ = 0.250. Since NMS axis are not always arranged in terms of order of effect, ordinations were also carried out using Principal Coordinates Analysis to confirm that the primary sources of variation were oriented on their appropriate axis of the NMS plots.

The significance of sample material (cecum vs. distal colonic pellets), sex, and access to GS on bacterial community composition and structure was tested using R by permutational multivariate ANOVA (perMANOVA; VEGAN v2.5-6) and by the non-parametric multi-response permutation procedure (MRPP; VEGAN v2.5-6; [43]). Indicator species analysis was performed using Indicspecies v1.7.8 with correction for false rate of discovery when appropriate. Alpha diversity was calculated using Phyloseq v1.30 and beta diversity was calculated using Betapart v1.51. LDA effect size (LEfSe) was determined using lefser v3.18. Packages were obtained from the comprehensive R archive network (CRAN; https://cran.r-project.org/).

Differences in weight gain, food intake, biochemistry measures and cytokine concentrations were determined by ANOVA (Statistica v13.1, Dell Statistica, Tulsa OK). It was noted in some instances that there was a large difference between the multiple R^2^ and the adjusted R^2^ when both sex and access to GS were included in the ANOVA analysis and the effect of sex had *p* values < 0.10. In these cases, the effect of access to GS was ignored and the effect of sex was tested using a t-test. When required, data were normalized following the method of Box and Cox [44]. Differences in total 16 S rRNA excretion were assessed using a general linear model with RS as the continuous predictor and sample source, sex and GS access as categorical predictors (Statistica v13).

## Results

### Food intake, body weight, fecal output, and biochemistry

**Fig. 1:**
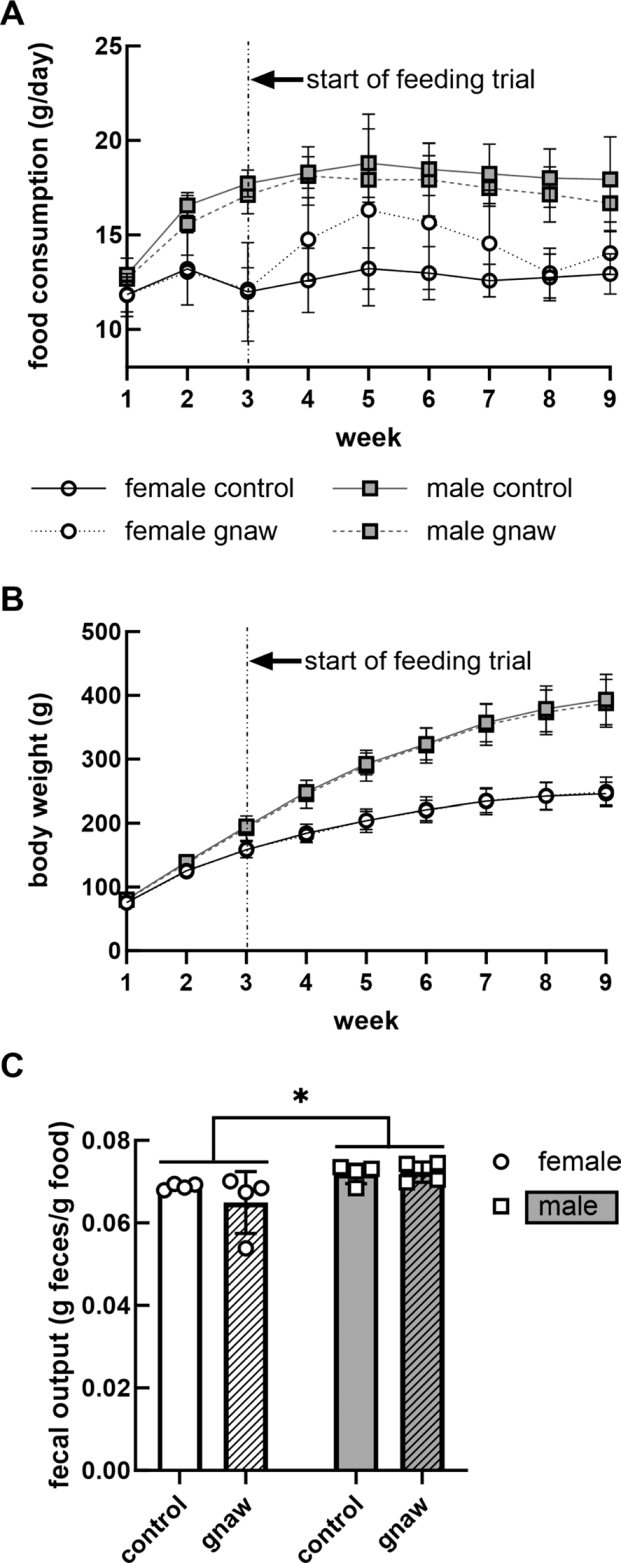
Food consumption as cage average (**A**; g/day/rat, *n* = 4) and total body weight (**B**; g, *n* = 8) over the 9-week study period, and fecal output (**C**; g feces/g food, *n* = 4) during the 2-week balance period. Error bars associated with points on the plot represent standard error of the means. * *p* = 0.03

Food intake values were calculated by cage and divided by two as the rats were pair-housed. Food intake differed by day (*p* < 0.0001) and sex (*p* = 0.0001), but was not affected by the introduction of GS into the cages (*p* = 0.65; Fig. 1A). Similar results were observed for body weight over the course of the experiment (Fig. 1B). Over the two week balance period, males excreted relatively more feces (0.072 ± 0.001 g feces/g food; *n* = 4 pairs) than females (0.067 ± 0.002 g feces/g food; *p* = 0.03; *n* = 4 pairs) but GS did not affect total fecal excretion (*p* = 0.42; Fig. 1C).

**Fig. 2:**
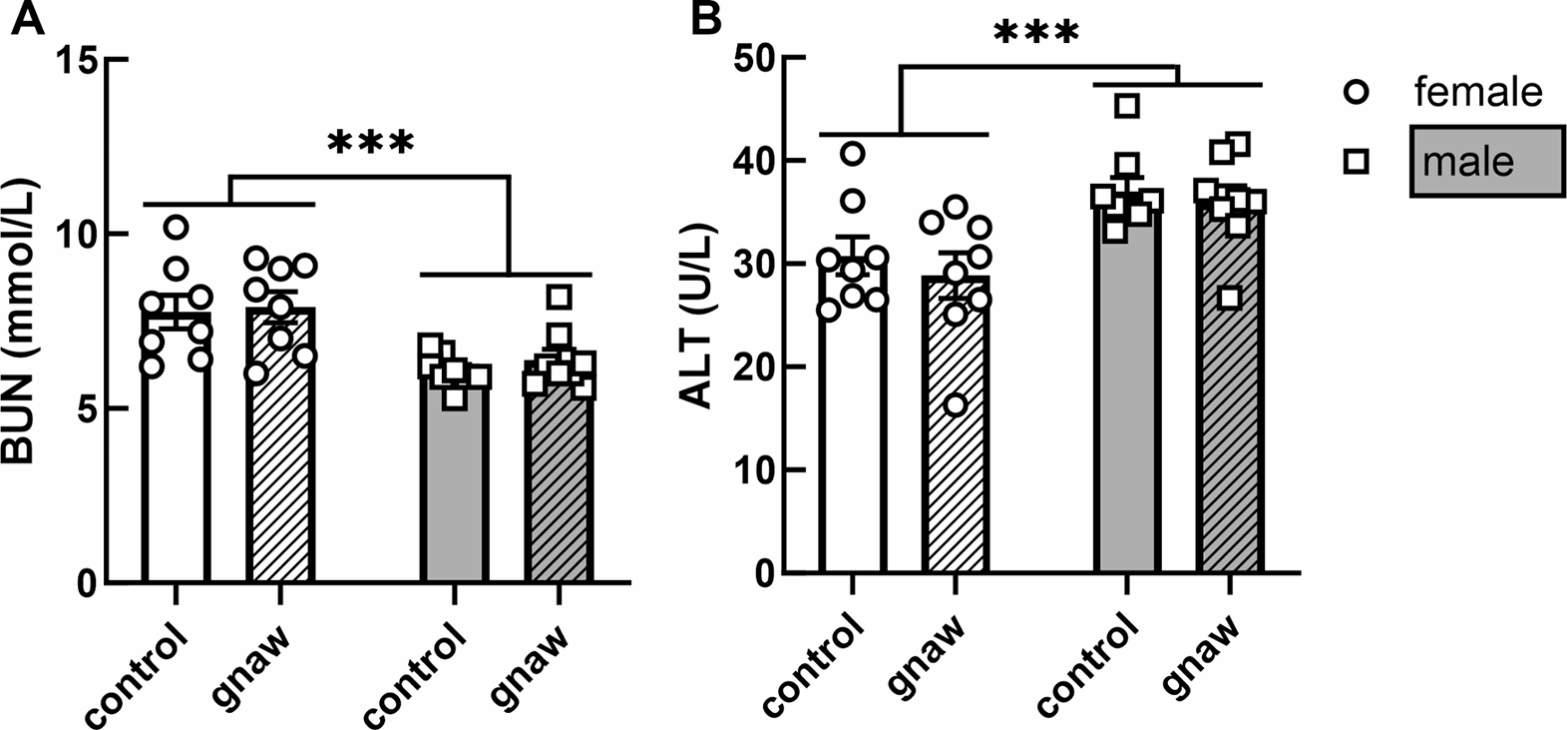
BUN (**A**; mmol/L) and ALT (**B**; U/L) concentrations in serum collected from female and male SD rats with or without access to GS. Females are represented by white bars with circles; males are represented by grey bars with squares, GS access is represented by hatching pattern. Error bars associated with histogram bars represent standard error of the means (*n* = 8). ***p < 0.001

BUN concentrations were higher in females (7.8 ± 0.3 mmol/L) when compared to males (6.2 ± 0.2 mmol/L; *p* < 0.001; Fig. 2A). On the other hand, serum ALT levels were higher in males (36.5 ± 1.1 U/L) vs. females (29.8 ± 1.4 IU/L; *p* < 0.001; Fig. 2B). No sex-dependent differences were observed for the other serum-measured liver-related enzymes LDH, ALP, and AST, which were also unaffected by access to GS.

### Cecal and fecal metabolites

Microbial fermentation profiles were affected by the inclusion of GS in a sex and location-specific manner (Table 1). Cecal SCFA concentrations were reduced in males with GS access relative to controls (*p* < 0.05). Sex-based differences (*p* < 0.05) were observed between cecal propionate and each of the BCFAs reported, but only when rats were given access to GS, and fecal acetate concentration was also affected differently between sexes (*p* < 0.05).

**Table 1:** Cecal and distal colon pellet short chain fatty acid concentrations and distribution

Location		Cecal				Distal colonic pellets			
Sex		Female		Male		Female		Male	
Condition		Control	GS	Control	GS	Control	GS	Control	GS
**SCFA**	Total (µmol/gww)*	41.1 ± 4.3	50.2 ± 6.5	53.2 ± 6.6	34.6^**a**^ ± 4.7	32.3 ± 5.4	25.6 ± 2.9	35 ± 3.9	27 ± 5.2
	Acetate (%)	58.6 ± 2.1%	64.0 ± 2.3%	62.2 ± 1.9%	55.1 ± 3%	60 ± 2.3%	63.4^**b**^ ± 1.3%	62 ± 1.7%	56.8^**b**^ ± 4%
	Propionate (%)	15.6 ± 0.5%	15.3^**b**^ ± 0.4%	16.2 ± 0.8%	17.9^**b**^ ± 0.5%	11.9 ± 0.6%	12.5 ± 1.1%	12.4 ± 1.1%	13.7 ± 1%
	Butyrate (%)	20.0 ± 1%	15.6 ± 1%	15.0 ± 0.6%	17.6 ± 2%	21.1 ± 2.1%	16.1 ± 1%	17.2 ± 1.3%	18.6 ± 3.2%
**BCFA**	Total (µmol/gww)*	1.3 ± 0.1	1.1^**b**^ ± 0.1	1.9 ± 0.2	1.9^**b**^ ± 0.1	1.4 ± 0.1	1.3 ± 0.1	1.7 ± 0.1	1.7 ± 0.2%
	Iso-butyric (%)	1.8 ± 0.1%	1.6^**b**^ ± 0.2%	2.0 ± 0.2%	3.0^**b**^ ± 0.3%	2.1 ± 0.2%	2.3 ± 0.3%	2.3 ± 0.2%	3 ± 0.5%
	Iso-valeric (%)	1.6 ± 0.1%	1.4^**b**^ ± 0.2%	2.0 ± 0.4%	3.1^**b**^ ± 0.4	2.7 ± 0.4%	2.9 ± 0.4%	2.7 ± 0.3%	4.5 ± 0.9%
	Valeric(%)	2.3 ± 0.1%	2.2^**b**^ ± 0.2%	2.3 ± 0.2%	3.2^**b**^ ± 0.4%	1.8 ± 0.1%	2 ± 0.2%	1.7 ± 0.2%	2.2 ± 0.3

* Total SCFA amount is shown as µmol/gww (gram of wet weight of cecal contents or colonic pellets), individual metabolites are shown as % of total SCFA

^**a**^Significantly different from control male control value (*p* < 0.05)

^**b**^Significant difference between sexes in GS groups, but not controls (*p* < 0.05)

### Cecal and distal colonic pellet community analysis

Bacterial 16 S rRNA content in cecal digesta and distal colonic pellets was assessed using the universal HDA1/HDA2 primers (13,19) and is expressed relative to total DNA present in the extraction to correct for potential differences in extraction efficiency of the community DNA. Fecal pellets collected from the balance study contained approximately twice the bacterial load when compared to cecal content values (3.3 ± 0.3 × 10^6^ vs. 1.8 ± 0.1 × 10^6^ copy number/ng community DNA). Taking into account total fecal excretion determined from the balance study, males excreted 1.8 times more bacteria than females (4.6 ± 0.6 vs. 2.6 ± 0.2 × 10^6^ (copy number/ng community DNA/g dry weight/d)). No effect of GS access was noted on 16 S rRNA copy number or excretion.

Community alpha diversity was assessed using four different measures at the family level. While no major differences were observed, and the non-parametric Chao1 was not different between groups (Table 2), Shannon diversity (which weights taxon richness more heavily (20); *p* = 0.027) and Simpson diversity (which favours taxon evenness more heavily (20); *p* = 0.001) were lower in distal colonic samples. In addition, female rats provided with GS had a lower Shannon diversity than those without GS (*p* < 0.05). Similar differences were observed at the genus level. Comparisons of beta-diversity among sample groupings showed a lower diversity in males (*p* = 0.03) but no effect of GS.

**Table 2:** Average alpha diversity measures (mean ± SEM) for bacterial communities at the family level

Sample	Sex	GS	Chao1	Shannon	Simpson	Fisher
All	All	All	34.6 ± 0.2	2.02 ± 0.03	0.800 ± 0.006	3.25 ± 0.02
Cecal			35.1 ± 0.3	2.07 ± 0.03	0.818 ± 0.006	3.29 ± 0.03
Distal colon			34.2 ± 0.4	1.96 ± 0.04^a^	0.781 ± 0.010^a^	3.21 ± 0.04
	Female		34.7 ± 0.4	2.01 ± 0.04	0.798 ± 0.010	3.25 ± 0.03
	Male		34.6 ± 0.3	2.02 ± 0.03	0.801 ± 0.008	3.26 ± 0.04
		Without	34.7 ± 0.4	2.07 ± 0.04	0.809 ± 0.009	3.25 ± 0.04
		With	34.6 ± 0.3	1.96 ± 0.03	0.790 ± 0.009	3.25 ± 0.03
Cecal		Without	35.2 ± 0.5	2.11 ± 0.05	0.825 ± 0.009	3.28 ± 0.04
Cecal		With	35.0 ± 0.4	2.02 ± 0.04	0.811 ± 0.008	3.29 ± 0.04
Distal colon		Without	34.1 ± 0.6	2.02 ± 0.06	0.791 ± 0.015^b^	3.21 ± 0.07^b^
Distal colon		With	34.2 ± 0.4	1.91 ± 0.05	0.771 ± 0.013^c^	3.21 ± 0.04^c^
	Female	Without	34.8 ± 0.6	2.12 ± 0.06	0.817 ± 0.013	3.25 ± 0.05
	Female	With	34.6 ± 0.4	1.90 ± 0.05^d^	0.779 ± 0.012	3.25 ± 0.04
	Male	Without	34.5 ± 0.6	2.01 ± 0.05	0.800 ± 0.012	3.26 ± 0.06
	Male	With	34.6 ± 0.4	2.03 ± 0.04	0.802 ± 0.011	3.26 ± 0.04

^**a**^Significantly different from cecal value (*p* < 0.05)

^b^Significantly different from cecal value without GS (*p* < 0.05)

^c^Significantly different from cecal value with GS (*p* < 0.05)

^d^Significantly different from female value without GS (*p* < 0.05)

**Fig. 3:**
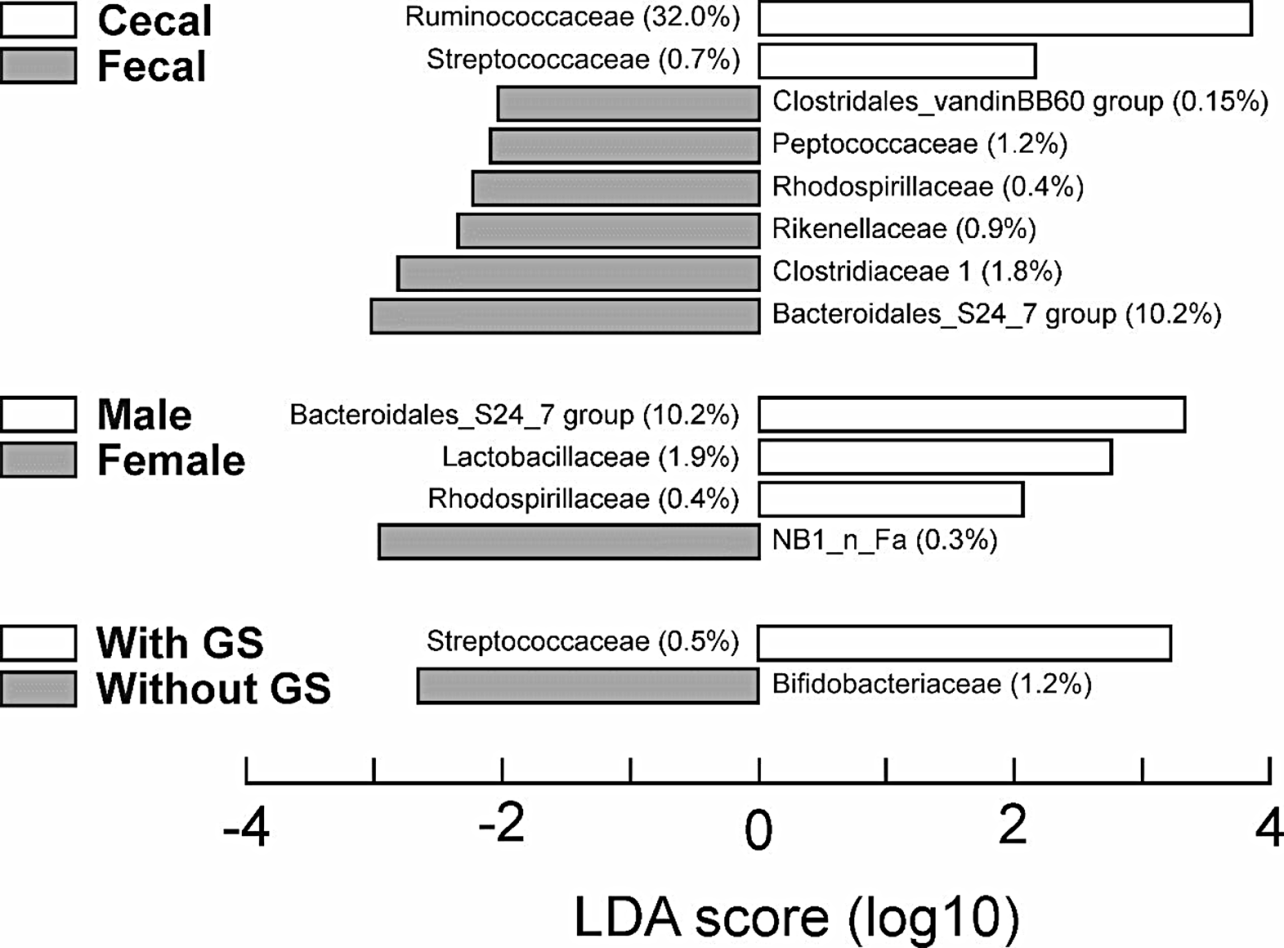
Linear discriminant analysis and Effect Size (LEfSe) analysis of bacterial family abundances for combined fecal and cecal samples. Values in parentheses show the percentage of the community associated with the named families

Analysis of bacterial community structure (assessed at the family level by PerMANOVA) showed an effect of sample source (cecal vs. distal colonic pellet material; *p* = 0.01) and a sex × GS access interaction (*p* = 0.01). Further analysis (by MRPP) of the interaction showed only a tendency towards an effect in females with GS (*p* = 0.08) demonstrating that the impact was minimal. Examination of differences in family abundances showed many changes related to sample source (Fig. 3) but fewer related to sex or access to GS. This was confirmed by a NMS plot overlaid with bacterial family abundances showing that the major difference between cecal and distal colonic samples was the proportion of Ruminococcaceae (data not shown). The importance of these differences can be assessed by taking into account the relative abundance of the bacterial families (Fig. 3). This showed that the two major identified changes occurred in the Ruminococcaceae and Bacteroidales S24-7 group. All other changes were relatively minor (Table 3).

**Table 3:** List of significant bacterial abundance changes as a function of source, sex and GS access at the family taxon level

Comparison	Bacterial Family	Community abundance (%)		Absolute Δ (Group 2 vs. Group 1)
		Group 1	Group 2	
**Cecal vs. colonic pellets**	Ruminococcaceae	26.84	36.88	10.04
	Bacteroidales S24-7 group	12.08	8.68	-3.4
	Christensenellaceae	1.37	2.21	0.84
	Clostridiaceae_1	2.18	1.24	-0.94
	Peptococcaceae	1.29	1.04	-0.25
	Rikenellaceae	1.03	0.74	-0.29
	Streptococcaceae	0.39	0.6	0.21
	Rhodospirillaceae	0.51	0.24	-0.27
	Clostridiales vadinBB60 group	0.23	0.06	-0.17
	Deferribacteraceae	0.124	0.045	-0.08
	Micrococcaceae	0.045	0.067	0.022
	Alphaproteobacteria Class	0.06	0.015	-0.05
**Males vs. Females**	Bacteroidales S24-7 group	8.72	12.15	3.43
	Lactobacillaceae	1.42	2.35	0.93
	Rhodospirillaceae	0.3	0.45	0.15
	NB1-n Order	0.39	0.23	-0.16
	Clostridiales Order	0.23	0.13	-0.1
	Desulfovibrionaceae	0.026	0.042	0.016
	Staphylococcaceae	0	0.0027	0.0027
	Thermoanaerobacteraceae	0.0002	0.0009	0.0007
**With/without GS**	Erysipelotrichaceae	4.27	1.8	-2.47
	Streptococcaceae	0.39	0.59	0.2
	Micrococcaceae	0.043	0.069	0.026

### Tissue cytokine profiles

Differential outcomes in tissue cytokine concentrations between rats with and without access to GS were observed in the liver, spleen, ileum and distal colon (Table 4). Liver IFN-γ concentrations were highest in rats with access to GS (*p* < 0.001), and liver IL-10 was higher in male rats with access to GS than males without (*p* < 0.01) and females with GS access (*p* < 0.001). Females with GS access (*p* < 0.01) and males without access (*p* < 0.01) displayed higher ileal IFN-γ concentrations than males with GS access. In the distal colon, CINC-1 concentrations were higher in both male and female rats without access to GS than those with access (*p* < 0.05). Four splenic cytokines showed differences with GS access, and the pattern was most consistent in females, where concentrations of three pro-inflammatory cytokines (IFN-γ, IL-1β, IL-6) were lower with GS access than without. Female rats without access to GS displayed higher splenic IFN-γ (*p* < 0.05) than females with GS access. Similarly, female rats without GS access had higher splenic IL-1β than females with access (*p* < 0.05) and males without access (*p* < 0.01). Female rats without GS access displayed higher splenic IL-6 concentrations than their corresponding GS group (*p* < 0.01), and females without GS access had higher splenic IL-10 than males housed in the same condition (*p* < 0.001).

**Table 4:** Tissue cytokine concentrations highlighting differences between female and male SD rats with or without access to GS

Cytokine	Tissue	Sex and GS Access	Concentration (pg/g) ± SEM, *n* = 8
**CINC-1**	liver	female, control	8.354 ± 0.6895++
		female, GS	7.493 ± 1.031++
		male, control	13.74 ± 1.535
		male, GS	10.06 ± 1.302
	cecum	female, control	296.2 ± 31.11+
		female, GS	362.8 ± 76.68+
		male, control	310.8 ± 72.08
		male, GS	311.8 ± 65.58
	distal colon	female, control	1306 ± 112.9
		female, GS	1242 ± 90.08*
		male, control	1564 ± 116.8
		male, GS	1197 ± 67.55*
**IFN-γ**	liver	female, control	14.20 ± 3.789
		female, GS	27.50 ± 3.972***
		male, control	13.23 ± 1.264
		male, GS	28.14 ± 4.536***
	spleen	female, control	2884 ± 673.2
		female, GS	1153 ± 134.9*
		male, control	1844 ± 304.9
		male, GS	1956 ± 669.3*
	ileum	female, control	1003 ± 169.8
		female, GS	885.8 ± 218.5^^
		male, control	580.8 ± 80.27
		male, GS	276.2 ± 71.16**
**IL-1β**	spleen	female, control	56.62 ± 3.897^^
		female, GS	42.21 ± 4.228*
		male, control	34.70 ± 5.426
		male, GS	46.48 ± 4.301
**IL-4**	liver	female, control	4.416 ± 0.4849+++
		female, GS	3.099 ± 0.3399+++
		male, control	5.941 ± 0.4938
		male, GS	5.991 ± 0.4639
**IL-6**	spleen	female, control	15.46 ± 1.095
		female, GS	10.78 ± 1.221*
		male, control	11.94 ± 0.7192
		male, GS	11.16 ± 0.9943
	cecum	female, control	5950 ± 745.1++
		female, GS	6908 ± 1783++
		male, control	3396 ± 631.0
		male, GS	3320 ± 1191
**IL-10**	liver	female, control	60.64 ± 3.289
		female, GS	54.28 ± 5.529^^^
		male, control	60.47 ± 5.461
		male, GS	80.07 ± 5.634**
	spleen	female, control	5350 ± 260.8^^
		female, GS	4168 ± 674.2
		male, control	3254 ± 256.2
		male, GS	4500 ± 639.9
	ileum	female, control	1703 ± 164.9^
		female, GS	1258 ± 181.6
		male, control	1021 ± 220.6
		male, GS	1503 ± 246.0
	cecum	female, control	2061 ± 407.2+
		female, GS	3089 ± 837.1+
		male, control	1394 ± 274.5
		male, GS	1726 ± 446.3
**Total TGF-β1**	spleen	female, control	200.3 ± 38.27+++
		female, GS	227.2 ± 11.57+++
		male, control	45.29 ± 10.04
		male, GS	20.66 ± 7.769
	distal colon	female, control	992.0 ± 101.8+
		female, GS	2257 ± 343.9+
		male, control	5394 ± 2572
		male, GS	4966 ± 2179
**TNF-α**	liver	female, control	23.90 ± 2.161++
		female, GS	22.17 ± 3.118++
		male, control	28.33 ± 2.649
		male, GS	34.11 ± 2.978

Data is expressed as mean ± standard error of the mean (SEM) in pg/g tissue

* significantly different from same sex control, *p* < 0.05; ** *p* < 0.01; *** *p* < 0.001

+ females significantly different from males regardless of gnaw stick access, *p* < 0.05; ++ *p* < 0.01; +++ *p* < 0.001

^ females significantly different from males in same EE group, *p* < 0.05; ^^ *p* < 0.01; ^^^ *p* < 0.001

For several tissue cytokines, sex-based differences were apparent, with limited or no impact of GS (also shown in Table 4). Males displayed higher liver concentrations of CINC-1 (*p* < 0.01), TNF-α (*p* < 0.01) and IL-4 (*p* < 0.001) than females. Female rats had higher splenic total TGF-β1 overall (*p* > 0.001) and higher ileal IL-10 (*p* < 0.01) in controls without access to GS. In the distal colon, males had higher concentrations of total TGF-β1 (*p* < 0.05). Females had higher cecal concentrations of CINC-1 (*p* < 0.05), IL-6 (*p* < 0.01) and IL-10 (*p* < 0.05) than males.

Overall, the inclusion of GS influenced IFN-γ, IL-1β, IL-6, IL-10 and CINC-1 concentrations in a location and sex-dependent manner amongst ileal, distal colon, splenic and liver tissues. While no consistent pattern of differences was observed, the GS added complexity to interpretation of sex-based differences for several tissue cytokines in SD rats. Concentrations of IL-17 F and sICAM-1 were also measured in these tissues; however, no significant differences were observed between access to GS or between sexes for these cytokines (data not shown). The cytokine profiles analyzed in the ileal Peyer’s patches and MLN also did not reveal sex-based differences or impacts of GS (data not shown).

## Discussion

Our results demonstrate that the inclusion of GS as part of an EE strategy can affect experimental outcomes relevant for analysis of the gut microbiota and immune system. Our study design included enriched cages for all rats with glass balls, background music and stainless steel housing, and the impact of access to GS as a single EE difference indicates their inclusion in experiments should be carefully considered depending on the planned measurements. Sex-specific differences between rats with access to GS were observed to impact bacterial fermentation profiles and contribute to minor differences in microbial community structure and in tissue cytokine concentrations of gastrointestinal and systemic tissues.

Including EE within animal cages has many potential benefits from animal welfare and experimental design perspectives [1, 2, 30]. However, it is important to consider that aggression may intensify in co-housed male mice with access to some forms of EE in their cages [20, 21], which could also impact experimental outcomes. Animal models are necessary for mechanistic studies of human health and disease, and it is important to consider all aspects of experimental design that can introduce variability or influence reproducibility. Potential factors contributing to differences in outcomes between studies include strain choice, animal supplier, diet composition, vivarium hygiene, housing conditions, handling, as well as individual lab practices and experimental designs. Ingestion of non-food EE components may also influence animal health and study outcomes [4]. While animal suppliers are usually reported in publications, authors often refer to “standard rodent chow” and “standard housing conditions” when describing experimental design, leading to open interpretation and potentially influencing reproducibility. These types of issues in methodology reporting have been raised in a recent mapping review, as has the need to consider enrichment of rat housing environments and potential for differences due to sex and strain [30]. Variations in describing EE can also lead to complications in meta-analyses. For example, it has been estimated that 50% of preclinical results are irreproducible due to incomplete reporting and variations in experimental designs [45, 46], and the importance of clearly defining and documenting EE in pre-clinical research studies has been previously emphasized [47].

The nylon chew bones used in the current study have been previously evaluated in SD rats and were not found to negatively impact body weight, food consumption and intestinal histology [48, 49]; however, these studies did not investigate effects on immune measures or gut microbial communities. A study comparing environmental enrichment with both rubber Kong^®^ chew toys and Nestlets^®^ nesting material reported lower levels of circulating adrenocorticotropic hormone and corticosterone in SD rats compared to control rats housed in similar cages without EE [50]. Male Wistar rats with access to either wooden chew sticks or plastic tubes show reduced rearing behaviour and increased fecal IgA concentrations, and the authors suggest that access to these EE tools may reduce hypothalamo-pituitary-adrenal axis activity [51]. While rubber chew toys, nesting materials, wooden chew sticks and plastic tunnels are considered safe for use in rodent studies, their inclusion should be reported and considered as a potential variable in studies examining steroid hormone concentrations, which influence numerous physiological activities and could further contribute to inconsistent experimental outcomes between studies.

Other types of EE have also been shown to affect experimental measures in different rodent models. We previously reported that housing Biobreeding rats in plastic shelters with maple wood chips significantly increased the Bacillota/Bacteroidota ratio (previously known as the Firmicutes/Bacteroidetes ratio) and the proportions of fecal acetate when compared to rats in wire-bottomed cages [31]. Enriched cages can have species-specific effects on hematological measures in different strains of mice (BALB/c, C57BL/6 and A/J), even when originating from the same provider and housed in the same specific pathogen free (SPF) conditions with a nest box, wood climbing bar and nesting material [52]. In another example, SPF BALB/cByJ mice housed with paper nesting material had increased eosinophil numbers and IL-13 concentrations in bronchiolar lavage fluid when compared to mice in cages with transparent plastic tunnels without nesting material and the non-enriched control group [53]. Additionally, mice housed in enriched cages containing Bio-Serv^®^ bio-huts, mouse igloos, tunnels and fast-track mouse wheels had improved survival curves and activated wound repair mechanisms, pericyte activation, and increased IgA secretion in a *Tcf4*^Het/+^*Apc*^Min/+^ mediated model of colon tumorigenesis [54]. A recent study examining effects of EE, in the form of access to a playpen area twice a week, on the liver tissue proteome of male SD rats found differences in certain proteins (Apolipoprotein A-I and Acyl-CoA 6-desaturase) involved in lipid metabolism [55]. While the authors focused on the liver as a key organ for assessing metabolic changes in response to EE, their novel approach illustrates the potential for proteomic and metabolomic analyses to elucidate mechanisms through which EE influences numerous physiological outcomes in rodents. Collectively, these findings further illustrate the varied effects of EE on experimental outcomes and the challenges in comparing studies conducted in enriched and non-enriched animal housing environments.

Our findings also indicate differences between sexes in the impact of EE on tissue cytokine profiles, including outcomes at local mucosal (ileum, cecum) and distal systemic (liver, spleen) locations. Significant differences in four splenic cytokine concentrations indicate an impact of EE at this systemic location. While no adverse health outcomes were observed, and the mechanisms underlying alterations to cytokine profiles of these four tissues in the presence of GS remains to be determined, these findings suggest GS inclusion may have an impact in rodent experiments measuring direct nutritional, immunological or endocrinological outcomes. While few studies have directly addressed sex-based differences in responses to EE, Hutchinson et al., (2005) reported a significant degree of thymic atrophy in female BALB/c mice with EE compared to unenriched controls, with no thymic atrophy in males [17]. Differences between sexes in EE-associated HPA axis activity have also been demonstrated in SD rats [7], and EE-associated reduction of CORT levels and fecal corticosterone metabolites is associated with increased splenic T and B cell numbers and enhanced secondary responses to influenza vaccination in male BALB/c mice [18]. Given the well-documented role of the HPA axis in modulating immune activity, future studies should address the potential connections between EE access, HPA axis activity and immune outcomes, and their interactions with gut microbiota activity.

Recently, differences in fecal metabolomic profiles of mice housed with or without EE have been reported, accompanied by differences in gut microbiota composition [56]. Male mice housed with EE had increased concentrations of formate and acetate in fecal water relative to non-EE controls, and mice fed (in the absence of EE) with these SCFA displayed several EE-associated behaviours and central neurochemical changes. While the mechanisms through which effects of EE are manifested at the level of the gut microbiota and immune system remain to be fully elucidated, our findings are in keeping with those of Marrocco et al. (2022) in demonstrating the impact of EE on gut microbial metabolic activity and the need to consider environmental cues in animal housing environments for research study design involving rodents [56]. Our findings also indicate that the impact of environmental cues provided through EE can differ between sexes.

It is often difficult to interpret the effect of individual EE treatments since the majority of studies include multiple interventions (exercise, socialization, toys, tunnels, food dispensers, number of co-housed animals, cage size, etc.). In the present study, rats were pair-housed with access to toys (glass balls), stainless steel shelters, and background music, and the only difference in EE between groups was access to GS. We focused on GS because of our interest in the interaction among diet, gut bacteria and immunological parameters; this requires not only strict control of diet ingredients, but also limiting access to potentially ingested substrates, including nesting materials and GS.

Our results show a relatively minor influence of GS on bacterial composition and diversity. This agrees with recent studies of mice where EE decreased Shannon diversity in young mice but this difference disappeared as the mice aged [57]. It also agrees with a study of Parkinson’s disease in mice where the Shannon diversity was similar in control and EE treated animals although some changes in gut bacteria were noted [58]. Similarly, there was a lack of effect of EE on diversity measurements in piglets [59, 60]. Interestingly, housed wild type mice had a higher diversity index when compared to field animals, further reflecting the complexity of interpreting effects of housing and EE on microbial community diversity [61].

Sex-based differences in EE outcomes in the context of nutrition-based studies have received little attention to date. Sex-specific effects have recently been observed in the effects of EE and dietary omega-3 supplementation with fish oil in adolescent Wistar rats [62]. In their study, EE was provided in the form of nesting materials, toys, acrylic tubes and a running wheel with changes in toys at two to three-day intervals. Rats receiving either fish oil or a soybean oil control were housed in either EE or regular cage conditions. An increase in immobility, considered to be a passive coping strategy to conserve energy in familiar environments, was observed in female rats fed fish oil and housed in regular conditions, but not when fish oil-supplemented females were housed in EE conditions. Measures of sociability were greater in female rats fed fish oil and housed in EE conditions, in contrast to males where the control soy oil diet promoted sociability. Reduced hippocampal glucocorticoid levels were also observed in EE-housed rats fed the control soybean oil diet and control-housed rats fed the fish oil diet, further illustrating the complex interactions between environment and diet, and the importance of investigating EE and sex-based differences in the context of nutrition studies.

Sex-based differences need to be continuously explored in animal studies. Females are frequently underrepresented in preclinical and clinical trials due to concerns about the impact of the estrous cycle on experimental variability. However, it has been shown that male hormones also fluctuate daily and can affect experimental measures as much or more than female hormone fluctuations [63]. Our findings indicate significant differences between sexes in the effect of access to GS on microbial metabolism and tissue cytokine concentrations. Given the observed differences in gastrointestinal parameters in this study and current knowledge about the sex-dependent effects of EE on brain development [64], it is possible that access to EE may also affect the gut-brain axis. The intestinal microbiota and metabolites produced can affect both gastrointestinal and blood-brain barriers which influence behavior and cognitive development [65]. This may have relevance for EE, as rats maintained in enriched environments following neonatal hypoxic ischemia were able to attenuate blood-brain barrier dysfunction compared to rats maintained in basic cages [66]. It remains to be elucidated whether exposure to GS influences intestinal permeability or differs between sexes.

Few studies have specifically examined effects of including nylon-based gnaw sticks for rats. A study testing the effect of including Nylabone nylon GS as EE for male SD rats on hyperphagia induced by orexin A, an appetite-stimulating neuropeptide, demonstrated that Nylabone GS inclusion had no significant impact on orexin-A-induced hyperphagia, suggesting that inclusion of GS does not influence short-term feeding behaviour [67]. In another study, strategies for pain assessment in chemotherapy-induced mucositis were compared in male SD rats treated with 5-fluorouracil (5-FU). 5-FU-treated rats gnawed the Nylabones included as EE to a greater extent than did saline-treated control rats, an unexpected behaviour that the authors suggested might serve as a distraction from pain [68]. Recently, changes in immune, metabolic and microbiota-related measures in rats have been observed following ingestion of microplastics (reviewed in [69]). Microplastic exposure has been reported to induce intestinal inflammation and increase intestinal permeability in rats [70], elevate hepatic IL-1β mRNA expression [71] and Th1-associated inflammatory activity [72], and to decrease expression of miRNA involved in gut barrier function [73]. Microplastic-induced changes in microbial community structure and metabolic activity have also been reported [70–72, 74], including altered serum concentrations of several microbial metabolites [75]. Taken together, these studies suggest that plastic-based GS used as EE may influence gut microbiota and immune-related measures with ingestion of minimal amounts of EE components potentially at levels not readily visible or detected by weighing. While we did not directly evaluate differences in GS ingestion or usage by weighing or visual examination, this study limitation could be addressed in future work to further explore differences in EE impact between sexes.

## Conclusion

We found that use of GS as an EE tool can influence gut microbiota and immune-related measures and can also lead to interactions in sex-based differences in this context. While future studies to further interrogate the impact of this type of EE on the immune system will be valuable to fully assess the extent of impact on immune activity and in determining the mechanisms involved, we believe these results indicate the importance of detailed reporting of EE conditions in rodent studies. We acknowledge that the experimental outcomes from rodents in complex and stimulating environments may be more consistently reproduced than from rodents in non-enriched environments. In order to fully elucidate the influence of EE on physiological parameters, direct comparisons must be made between studies designed with and without different EE strategies. Although rubber and nylon chew toys have been deemed safe for rodent studies, their inclusion as EE should be carefully deliberated in nutritional and immunological studies given that GS have the potential to influence experimental outcomes, an important consideration for pre-clinical studies.

## Electronic supplementary material

Below is the link to the electronic supplementary material.

Supplementary Material 1

Supplementary Material 2

Supplementary Material 3

Supplementary Material 4

## Data Availability

All sequencing data files have been included in this submission as supplemental data.

## Abbreviations

ALP: Alkaline phosphate
ALT: Alanine aminotransferase
AST: Aspartate aminotransferase
BCFA: Branched chain fatty acid
BDNF: Brain-derived neurotrophic factor
BUN: Blood urea nitrogen
CINC-1: Cytokine-induced neutrophil chemoattractant 1
CORT: Corticosterone
EE: Environmental enrichment
ELS: Early life stress
GDNF: Glial-derived neurotrophic factor
GS: Gnaw sticks
gww: Gram of wet weight
HI: Hypoxic-ischemic
IFN-γ: Interferon-γ
IL: Interleukin
LDH: Lactate dehydrogenase
LDA: Linear discriminant analysis
LEfSe: LDA effect size
MIA: Maternal immune activation
MRPP: Multi-response permutation procedure
NK: Natural Killer cell
NMS: Non metric multidimensional scaling
SCFA: Short chain fatty acid
sICAM-1: Soluble intercellular adhesion molecule-1
SD: Sprague-Dawley
TGF-β1: Transforming growth factor-beta 1
TNF-α: Tumour necrosis factor-α
